# Low Birth Weight Intensifies Changes in Markers of Hepatocarcinogenesis Induced by Fructose Consumption in Rats

**DOI:** 10.3390/metabo12100886

**Published:** 2022-09-21

**Authors:** Lorena de Souza Almeida, Caio Jordão Teixeira, Carolina Vieira Campos, Laís Guadalupe Casaloti, Frhancielly Shirley Sodré, Vinícius Cooper Capetini, Andressa Godoy Amaral, Tanyara Baliani Payolla, Lucas Carminatti Pantaleão, Gabriel Forato Anhê, Silvana Bordin

**Affiliations:** 1 Department of Pharmacology, Faculty of Medical Sciences, State University of Campinas, 105 Alexander Flemming St., Campinas 13083-881, SP, Brazil; 2 Department of Physiology and Biophysics, Institute of Biomedical Sciences, University of Sao Paulo, 1524 Prof. Lineu Prestes Ave., ICB 1, Sao Paulo 05508-000, SP, Brazil; 3 Wellcome-MRC Institute of Metabolic Science-Metabolic Research Laboratories, University of Cambridge, Cambridge CB2 0QQ, UK

**Keywords:** intrauterine growth restriction (IUGR), fructose, hepatic growth restriction, non-alcoholic steatohepatitis (NASH), hepatocellular carcinoma (HCC)

## Abstract

Intrauterine growth restriction (IUGR) due to fetal exposure to glucocorticoid excess results in metabolic inflexibility and hepatic steatosis upon nutritional stress during adulthood. We previously demonstrated that rats born to dexamethasone (DEX)-treated mothers developed hepatic steatosis when exposed to 10% fructose solution during adult life. Persistent triacylglyceride (TAG) accumulation in the liver, in turn, is a feature of non-alcoholic fatty liver disease (NAFLD), which serves as a risk factor for non-alcoholic steatohepatitis (NASH) and hepatocellular carcinoma (HCC). In the present study, we demonstrate that the combination of IUGR and fructose treatment during adulthood also results in increased hepatic myeloperoxidase (MPO) activity, AKT phosphorylation and serum aspartate transaminase. Growth-restricted rats also presented reduced hepatic TRIB3 and GADD45a after fructose treatment. Other markers of cell proliferation, such as Cyclin D, PCNA, Hgf and Hspa4/Hsp70 expression and the number of Ki-67 positive cells, were all increased in the liver of growth- restricted rats treated with fructose. On the other hand, the combination of IUGR and fructose treatment during adult life reduced the levels of IGF-1. In conclusion, our data indicate that after exposure to fructose, adult rats subjected to dexamethasone-induced IUGR display exacerbated molecular changes in markers of NASH and HCC.

## 1. Introduction

Non-alcoholic fatty liver disease (NAFLD) is characterized by excessive fat accumulation in the liver that predisposes to non-alcoholic steatohepatitis (NASH). Along with alcohol consumption and viral infections, NASH is a leading cause of cirrhosis and hepatocellular carcinoma (HCC) [1]. Recent epidemiological studies suggest that NAFLD patients may develop HCC as a consequence of both cirrhotic steatohepatitis and from non-cirrhotic malignant transformation of hepatocellular adenoma (HCA) into HCC [1,2,3,4].

Population-based studies collected consistent evidence showing that nutritional habits, such as excessive consumption of artificially sweetened soft drinks, are associated with increased risk for NAFLD independently of body weight gain [5,6,7]. In experimental contexts, liver fat accumulation is induced in rodents exposed to liquid fructose or fructose-enriched diets [8,9,10].

Besides the nutritional environment during adult life, it has been demonstrated that the risk of NASH and NAFLD is influenced by birth weight. Children born with both low and high birth weights are predisposed to the development of NAFLD [11,12,13]. We previously demonstrated that rats born with low birth weight caused by maternal exposure to dexamethasone (DEX) are also predisposed to hepatic steatosis, as they fail to mobilize liver triacylglycerides (TAGs) during a prolonged fasting period [14]. Notably, we recently described that both low birth weight and liquid fructose consumption during adulthood interact to exacerbate hepatic steatosis in adult rats [15].

Although we reported that the metabolic effects of fructose consumption are intensified in rats born to DEX-treated mothers, it remains to be clarified whether these two factors interact to modulate markers that predispose to NASH and HCC. With the aim of addressing this issue, we have presently evaluated multiple molecular markers that mediate the origin of HCC and NASH in the liver of rats with low birth weight that were subjected to fructose treatment during adult life.

## 2. Materials and Methods

### 2.1. Animals and Experimental Design

Three-week-old nulliparous male and female Wistar rats were kept under a 12 h light–dark cycle at 22 ± 2 °C with ad libitum access to rodent standard chow and water. Upon reaching 12 to 15 weeks of age, two female rats were housed in individual cages with one male for 3 days. The concomitant presence of spermatozoa and estrous cells in a vaginal lavage indicated day 0 of gestation.

Pregnant rats were treated with dexamethasone as previously described [15,16]. Briefly, pregnant rats were randomly assigned to two experimental conditions. Half of the pregnant rats remained untreated while the other half received dexamethasone (DEX) (0.1 mg/kg body mass/day; Achê Pharmaceutical Laboratories, Guarulhos, SP, Brazil) diluted in the drinking water from the 14th to the 19th day of pregnancy. Litter sizes were randomly culled to eight per mother at birth.

At eight weeks of age, male offspring born to DEX-treated mothers were assigned to receive water (DEX group) or a 10% fructose solution (DEX+fructose group). Male offspring born to untreated mothers were also assigned to receive either water (CTL group) or 10% fructose solution (fructose group). Treatment with fructose lasted for 8 weeks. All offspring received standard chow ad libitum from the weaning until the experiment day.

### 2.2. Analysis of Blood Parameters

The quantification of glucose, aspartate transaminase activity (AST), triacylglycerides, total cholesterol and HDL cholesterol were performed with the aid of commercially available kits (Labtest Diagnóstica SA, Lagoa Santa, MG, Brazil; LaborLab, Guarulhos, SP, Brazil).

IGF-1 plasma levels were measured by the Enzyme-Linked ImmunoSorbent Assay (ELISA) method, using the commercial IGF-1 Rat ELISA Kit (Thermo Scientific, Waltham, MA, USA, cat. number: ERIGF1) following the manufacturer’s instructions. Plasma samples were diluted to 1000-fold in appropriate diluents and assayed in duplicate.

### 2.3. Analysis of Tissue Parameters

Liver samples were quickly removed, washed with ice-cold PBS, snap-frozen with liquid nitrogen, and ground in a mortar. Powdered samples were stored in a deep freezer at −80 °C for subsequent uses.

Lipid extraction was performed as previously described [17]. Briefly, powdered samples were homogenized in CHCl_3_:methanol solution (2:1, *v/v*) and transferred to sealed glass tubes that were incubated overnight at 4 °C under gentle shaking. After incubation, samples were added to 0.6% NaCl solution and centrifuged at 2000× *g* for 20 min. The organic layer was removed and dried in an Eppendorf Vacuum Concentrator Plus (Eppendorf, Hamburg, Germany). The lipids were diluted in isopropanol and quantified using commercially available kits.

To determine the levels of myeloperoxidase (MPO), 100 mg of liver samples was homogenized in 1 mL of extraction buffer (5 mM K_2_PO_4_, pH 6.0 + 0.5% HTBA) and incubated for 2 h at 60 °C for inactivation of endogenous catalase. Samples were then centrifuged at 10,000× *g* for 5 min at 4 °C. Supernatants were collected and added to a 96-well plate with MPO substrate (K_2_PO_4_, 5 mM + H_2_O_2_ 0.0005% pH 6 + o-dianisidine chromophore agent (ODN)). Absorbance was determined for 10 min (reads every 50 s) at 460 nm to provide a kinetic curve of substrate consumption. The resulting curves were transformed into area under the curve (AUC) for final analysis.

Total RNA was extracted using Qiazol reagent (Qiagen, Germantown MD, USA) from approximately 100 mg of tissue and subsequently used for reverse transcription with random primers. The primer sequences and accession numbers were as follows: Gadd45a (NM_024127) sense 5′-TCAACATCCTGCGGGTCAGC-3′ and antisense 5′-TGTGGGTTCGTCACCAGCAC-3′; Trib3 (NM_144755) sense 5′-GTTGGTGCTGGAGAACCTGGAG-3′ and antisense 5′-AGAGTCCTGGAACGGGTATCGG-3′; Hgf (NM_017017) sense 5′-TGCAGTCAGCACCATCAAGGC-3′ and antisense 5′-GGACGATTTGGGATGGCACATC-3′; Hspa4 (NM_031971) sense 5′-GAGATCATCGCCAACGACCAG-3′ and antisense 5′-CTCGCCCTTGTAGTTCACCTGC-3′; Rpl37a (X14069) sense 5′-CAAGAAGGTCGGGATCGTCG-3′ and antisense 5′-ACCAGGCAAGTCTCAGGAGGTG-3′. Values of mRNA expression were normalized using the internal control gene Rpl37a [15]. The relative expressions for each gene were calculated by the 2-DDCT method.

Powdered liver samples of approximately 100 mg were processed for Western blotting as previously described [18]. The nitrocellulose membranes for WB were stained with Ponceau S before incubation with blocking solution and the primary antibodies. The stained membranes were allowed to dry at room temperature, scanned, and subjected to optical density (OD) quantification. All the lanes (from the top to the bottom) of labeled proteins were scanned to better represent the total amount of protein loaded in the gel. Subsequently, these values were used to normalize OD data of the target proteins detected in the respective membranes. The primary antibodies used were as follows: anti-MKP-1 (Santa Cruz Biotechnology, Santa Cruz, CA, USA; cat. # sc370), anti-phospho-ERK 1/2 (Cell Signaling Technology, Beverly, MA, USA; cat. #9102), anti-phospho-AKT Ser473 (Santa Cruz Biotechnology; cat. # sc7985), anti-Cyclin D1 (Santa Cruz Biotechnology; cat. # sc8396), anti-PCNA (Santa Cruz Biotechnology; cat. # sc56), anti-Ki-67 (Spring Bioscience, Pleasanton, CA, USA; cat. # M3064).

### 2.4. Immunohistochemistry (IHC)

Liver immunoperoxidase reaction was carried out as previously described [16]. Briefly, liver samples were removed, immersed in 4% paraformaldehyde formalin fixative solution for 24 h, and embedded in paraffin. Five-micron serial sections were mounted onto aminopropyltriethoxysilane-coated glass slides. Sections were deparaffinized, rehydrated and washed with 0.05 M Tris buffer solution (TBS) at pH 7.4. Subsequently, for antigen retrieval, sections were treated with 0.01 M Tris-EDTA buffer containing 0.05% Tween-20 (pH 9.0) for 24 min at 98 °C. After inactivation of endogenous peroxidase (0.3% hydrogen peroxide solution) and blocking of nonspecific antibody binding (5% BSA solution containing 0.1% Tween-20), sections were incubated with rabbit monoclonal anti-Ki-67 primary antibody (1:60) diluted in TBS containing 3% BSA overnight at 4 °C. Subsequently, sections were washed with TBS and incubated with HRP-conjugated anti-rabbit IgG (Nichirei Bioscience, Tokyo, Japan; cat. no. 414191F) for 2 h at room temperature. For detection of Ki-67 positive cells, a 3.3′ diaminobenzidine solution (DAB; Sigma Aldrich, St Louis, MO, USA, cat. no. D4293) was employed for 5 min. All slides were counterstained with Harris Hematoxylin and mounted for observation by microscopy.

Images were acquired using a light microscope OPTIKA ITALY B-1000 Series (OPTIKA S.r.l., Ponteranica, BG, Italy) equipped with a digital camera under a ×20 objective. Three sections from different parts of each sample (200 μm apart; from 3 to 4 animals per group) were analyzed. From each section, between 15 and 20 fields were acquired, and the Ki-67 positive cells per high power field were recorded [19]. Data are represented by the average of Ki-67 positive cells obtained in each section.

### 2.5. Statistical Analyses

Parametric distribution of the data was assessed with the Shapiro test. Data were compared using two-way ANOVA, followed by a Tukey’s multiple-comparison test. The factors for the two-way ANOVA were in utero exposure to DEX and treatment with 10% fructose during adulthood. When making comparisons between two groups, the unpaired Student’s t-test was used. Statistical analyses were made with GraphPad Prism software version 8.4.3 (GraphPad Software, Inc., San Diego, CA, USA). All results are presented as the means ± standard error of the mean (SEM). Results with *p* values lower than 0.05 were considered significant.

## 3. Results

### 3.1. Rats Born to DEX-Treated Mothers Display Reduced Liver Weight at the First Day of Life

**Figure 1:**
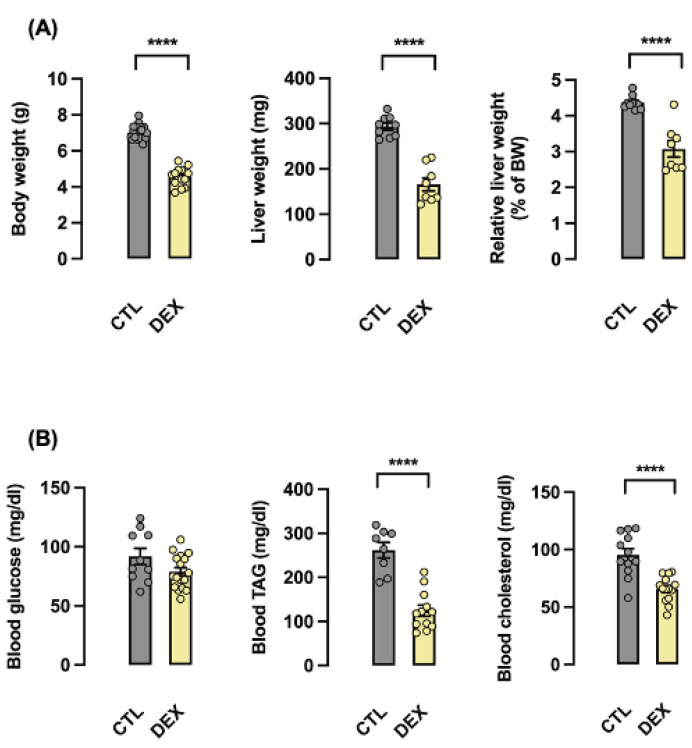
**Maternal dexamethasone treatment induced hepatic growth restriction and altered biochemical parameters in the offspring in early life.** Body mass, liver mass and relative liver mass of rats born to DEX-treated mothers (DEX) and untreated mothers (CTL) were assessed at the first day of life (**A**). Blood glucose, triacylglycerol (TAG) and total cholesterol were also assessed at the first day of life (**B**). Data are presented as the mean ± SEM; **** *p* < 0.0001 vs. CTL. In (**A**), offspring’s total body mass (CTL: *n* = 19; DEX: *n* = 14); total liver mass and relative liver mass (% of total body mass) (CTL: *n* = 10; DEX: *n* = 8). In (**B**), blood glucose (CTL: *n* = 10; DEX: *n* = 17), TAG (CTL: *n* = 8; DEX: *n* = 12) and total cholesterol (CTL: *n* = 12; DEX: *n* = 14).

Our initial approach was to determine the effects of in utero exposure to DEX in the offspring during early neonatal life. As previously demonstrated [20,21], rats born to DEX-treated mothers have reduced birth weight (34% lower than CTL; *p* < 0.0001). In utero exposure to DEX also led to reduced absolute and relative liver weight (respectively, 41 and 30% lower than CTL; *p* < 0.0001) (Figure 1A). Circulating TAG and total cholesterol levels, but not glucose, were lower in neonatal rats born to DEX-treated mothers (respectively, 53 and 31% lower than CTL; *p* < 0.0001) (Figure 1B).

### 3.2. Rats Born to DEX-Treated Mothers Display Lower Corticosterone Levels and Increased Hepatic ERK1/2 Phosphorylation

**Figure 2:**
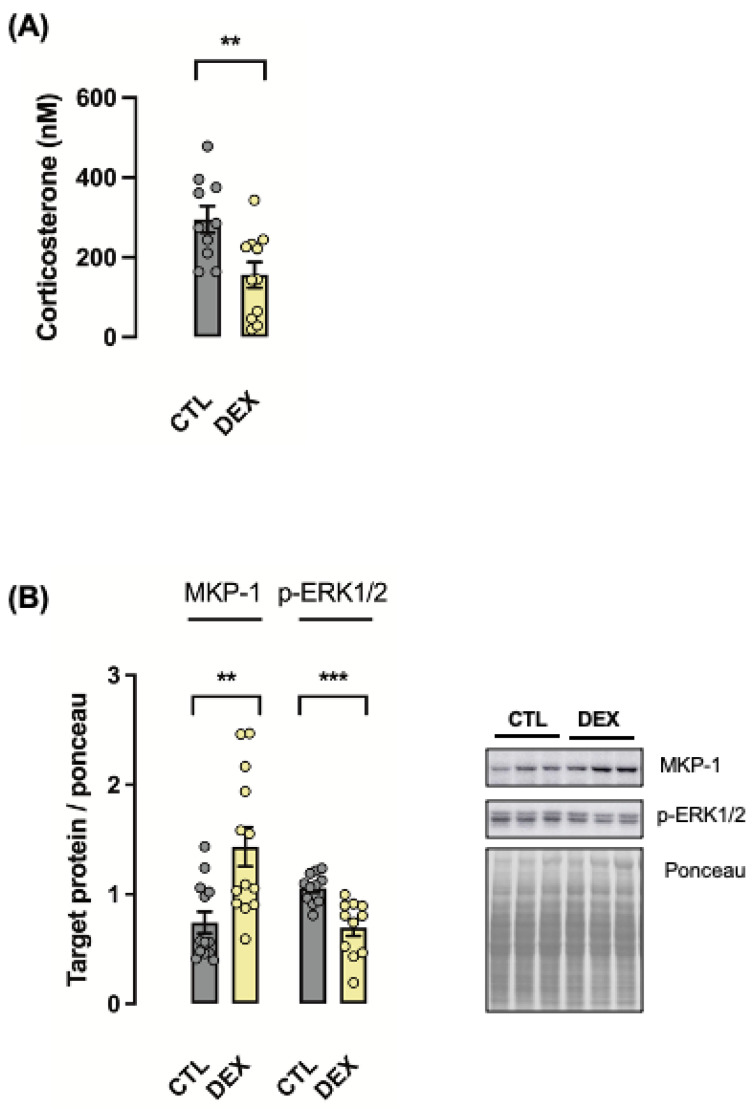
**Maternal dexamethasone treatment suppressed fetal hypothalamic**–**pituitary**–**adrenal (HPA) axis and increased MKP1 activity in the offspring liver in early life.** Blood samples were collected at the first day of life to measure corticosterone levels (**A**). Liver fragments were removed from rats born to DEX-treated mothers (DEX) and untreated mothers (CTL) at the first day of life and processed for Western blot detection of MAPK-1 (Mitogen-Activated Protein Kinase 1) and its target phosphorylated ERK1/2 (Extracellular Signal-Regulated Kinase 1 and 2). Band intensities were normalized by the signal from Ponceau staining. Data are presented as the mean ± SEM; ** *p* < 0.01; *** *p* < 0.0001 vs. CTL. In (**A**), CTL: *n* = 10; DEX: *n* = 11. In (**B**), *n* = 12 for both groups.

As a readout for neonatal hypothalamus–hypophysis–adrenal axis suppression, exposure to DEX during late pregnancy reduced corticosterone levels at the first day of life (47% lower than CTL; *p* < 0.01) (Figure 2A). The ERK1/2 signaling pathway, which was described to be involved in hepatic cellular proliferation, was also modulated by in utero exposure to DEX. An increase in the expression of the ERK1/2 phosphatase MKP-1 (89% higher than CTL; *p* < 0.01) with a reciprocal decrease in ERK1/2 phosphorylation (30% lower than CTL; *p* < 0.001) was found in the liver of neonatal rats born to DEX-treated mothers (Figure 2B).

### 3.3. Exposure to DEX during Fetal Life Retards the Increase in Circulating TAG Induced by Fructose in Adult Rats

**Figure 3:**
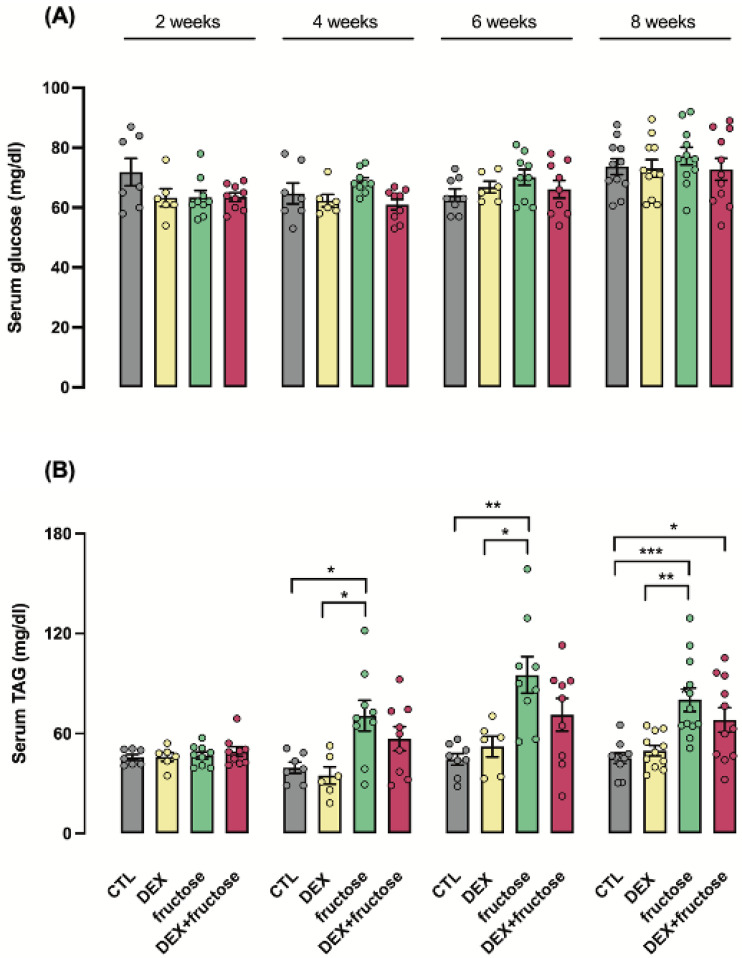
**Time course of biochemical parameters (glucose and TAG) in adult rats exposed or not to dexamethasone in utero.** At eight weeks of life, male offspring of dexamethasone-treated and untreated dams were divided into two additional groups that were either kept with tap water or 10% fructose (*w*/*v*) solution ad libitum for the next 8 weeks. The four groups were named as follows: offspring born to untreated mothers that received tap water during adult life (CTL); offspring born to DEX-treated mothers that received tap water during adult life (DEX); offspring born to CTL mothers that received 10% fructose during adult life (fructose); and offspring born to DEX-treated mothers that received 10% fructose during adult life (DEX+fructose). Biochemical parameters were evaluated at 2, 4, 6 and 8 weeks in animals that consumed fructose and age-matched rats consuming tap water. Systemic blood samples were collected to measure glucose (**A**) and triacylglycerol (**B**). Data are presented as the mean ± SEM; * *p* < 0.05; ** *p* < 0.01; *** *p* < 0.001 vs. the group indicated by the bracket. *n* = 6 to 12 in (**A**,**B**).

Fasting glucose levels were not changed over the course of fructose treatment of adult rats born to mothers that were either treated or not treated with DEX during pregnancy (Figure 3A). Circulating TAG levels were increased by fructose treatment of rats born to untreated mothers. This increase was detected as early as the fourth week of treatment (89% higher than CTL; *p* < 0.05) and lasted until the eighth week of treatment (78% higher than CTL; *p* < 0.001). On the other hand, the increase in TAG levels induced by fructose in rats born to DEX-treated mothers was only detected at the eighth week of treatment (51% higher than CTL; *p* < 0.05) (Figure 3B).

### 3.4. Exposure to DEX during Fetal Life Interacts with Fructose Treatment to Reduce HDL Cholesterol in Adult Rats

**Figure 4:**
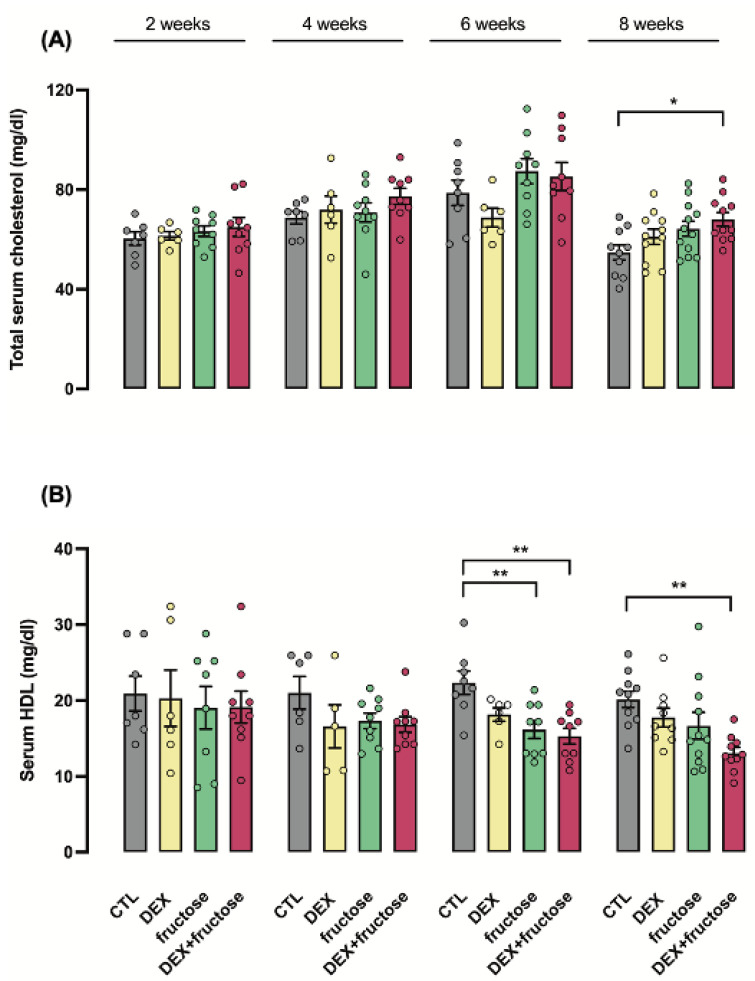
**Time course of biochemical parameters (total cholesterol and HDL) in adult rats exposed or not to dexamethasone in utero.** Blood samples of the four groups (described in Figure 3) were also used to measure total cholesterol (**A**) and high-density lipoprotein (HDL) (**B**). Data are presented as the mean ± SEM; * *p* < 0.05 and ** *p* < 0.01 vs. the group indicated by the bracket. *n* = 6 to 12 in (**A**,**B**).

Fructose treatment increased total cholesterol after eight weeks of treatment exclusively in rats exposed to DEX during fetal life (24% higher than CTL; *p* < 0.05) (Figure 4A). HDL cholesterol was reduced after six weeks of fructose treatment in adult rats born to mothers that were either treated or not treated with DEX during pregnancy (respectively, 27 and 32% lower than CTL; *p* < 0.01). This reduction was sustained up to the eighth week of treatment only in the Fructose–DEX group (35% lower than CTL; *p* < 0.01) (Figure 4B).

### 3.5. Exposure to DEX during Fetal Life Interacts with Fructose Treatment to Stimulate Parameters of NASH

**Figure 5:**
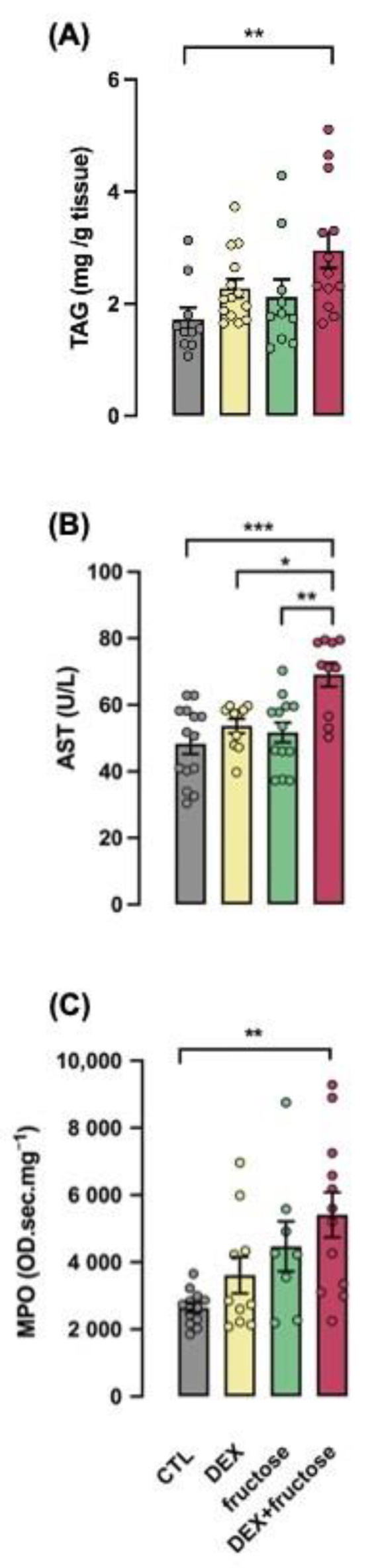
**Markers of NASH increased in rats born to DEX-treated mothers and that chronically consumed fructose in adult life.** Blood and liver samples of adult rats supplemented (fructose and DEX+fructose groups) or not (CTL and DEX groups) with fructose for 8 weeks were collected for analysis of hepatic content of triglycerides (TAGs) (**A**), circulating aspartate aminotransferase (AST) (**B**) and hepatic levels of myeloperoxidase (MPO) (**C**). Data are presented as the mean ± SEM; * *p* < 0.05; ** *p* < 0.01; *** *p* < 0.001 vs. the group indicated by the bracket. *n* = 10 to 13 for TAGs (**A**), *n* = 10 to 14 for AST (**B**) and *n* = 8 to 12 for MPO.

Hepatic TAG content, aspartate aminotransferase (AST) and myeloperoxidase (MPO) activity in the liver were assessed as markers of NASH. All these parameters were increased by eight weeks of fructose treatment exclusively in rats born to DEX-treated mothers (respectively, 76, 44 and 105% higher than CTL; *p* < 0.01, *p* < 0.001 and *p* < 0.01) (Figure 5A–C).

### 3.6. Signaling Pathways Involved in Cell Proliferation Are Exclusively Increased in Fructose-Treated Rats Exposed to DEX in Utero

**Figure 6:**
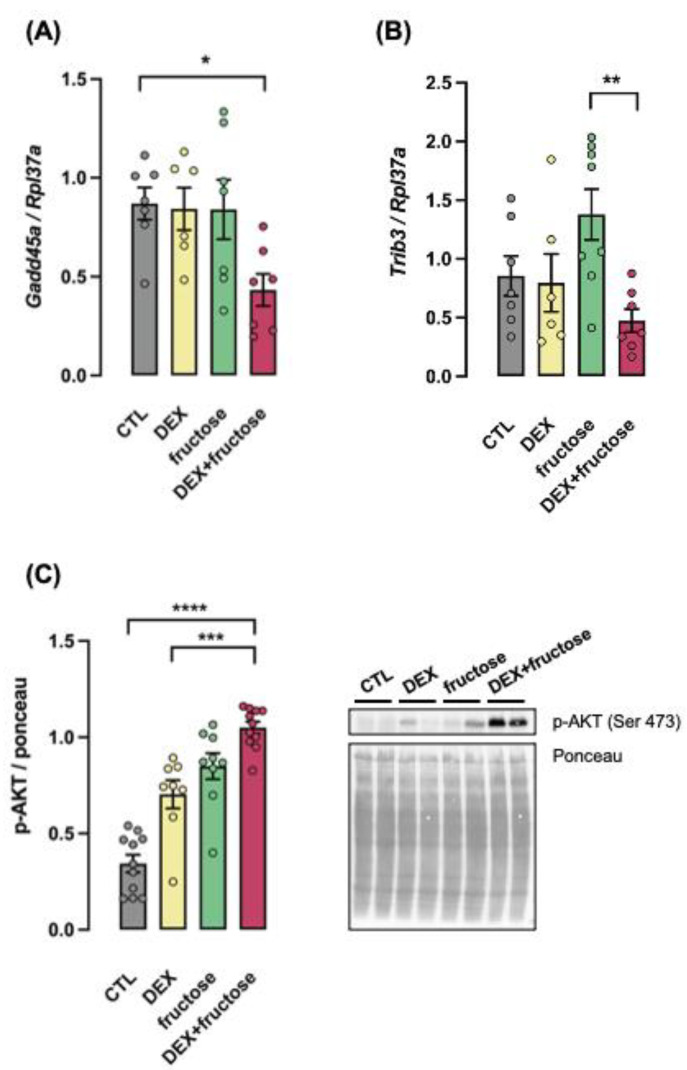
**Fructose consumption induced changes in HCC in the liver of rats born to DEX-treated mothers.** Liver fragments were removed from adult rats supplemented (fructose and DEX+fructose groups) or not (CTL and DEX groups) with fructose for 8 weeks. One set of samples was processed for RNA extraction and qPCR detection of Gadd45a (**A**) and Trib3 (**B**) expressions. Another set of samples was processed for Western blot detection of phosphorylated AKT (**C**). In the latter, band intensities were normalized by the signal from Ponceau staining. Data are presented as the mean ± SEM; * *p* < 0.05, ** *p* < 0.01, *** *p* < 0.001 and **** *p* < 0.001 vs. the group indicated by the bracket. In (**A**,**B**), *n* = 6 to 8; in (**C**), *n* = 8 to 11.

The mRNA expression of the growth-arresting protein Gadd45a and the AKT inhibitor Trib3 were reduced exclusively in the liver of rats born to DEX-treated mothers (respectively, 51% lower than CTL and 66% lower than fructose group; *p* < 0.05 and *p* < 0.01) (Figure 6A,B). In parallel to the reduction in hepatic Trib3 expression, increased AKT phosphorylation was significantly increased in the Fructose–DEX group (194% higher than CTL; *p* < 0.0001) (Figure 6C).

### 3.7. Markers Associated with HCC Are Exclusively Increased in Fructose-Treated Rats Exposed to DEX in Utero

**Figure 7:**
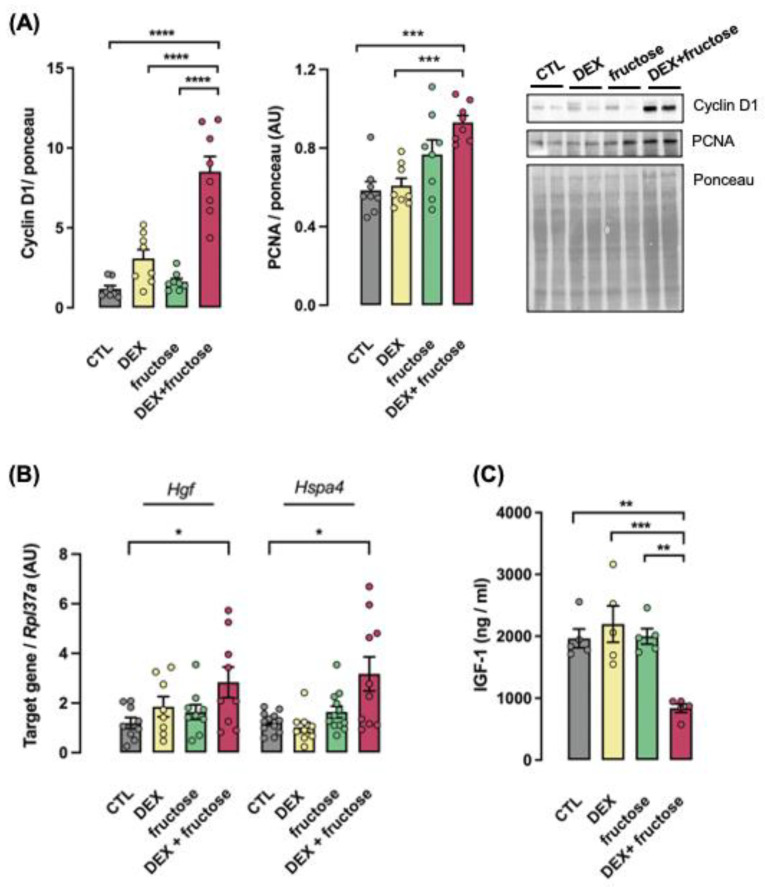
**Markers of HCC increased in rats born to DEX-treated mothers and that chronically consumed fructose in adult life.** Liver and blood samples were collected from adult rats supplemented (fructose and DEX+fructose groups) or not (CTL and DEX groups) with fructose for 8 weeks. Liver samples were used for immunoblotting detection of Cyclin D1 and PCNA (**A**), and qPCR detection of Hgf and Hspa4/Hsp70 expressions (**B**). Band intensities of immunoblotting experiments were normalized by the signal from Ponceau staining. Blood samples were used to measure circulating IGF-1 (**C**). Data are presented as the mean ± SEM; * *p* < 0.05, ** *p* < 0.01, *** *p* < 0.001 and **** *p* < 0.001 vs. the group indicated by the bracket. In (**A**), *n* = 8; in (**B**), *n* = 8 to 12; in (**C**), *n* = 5.

The protein levels of Cyclin D1 and proliferating cell nuclear antigen (PCNA), as well as the mRNA expression of Hepatocyte growth factor (Hgf) and Heat shock protein family A, member 4 (Hspa4), were increased only in the liver of fructose-treated rats that were born to mothers that received DEX during pregnancy (respectively, 608, 60, 177 and 167% higher than CTL; *p* < 0.0001, *p* < 0.001 and *p* < 0.05) (Figure 7A,B). The circulating levels of IGF-1 were only reduced in the Fructose–DEX rats (57% lower than CTL; *p* < 0.01) (Figure 7C).

**Figure 8:**
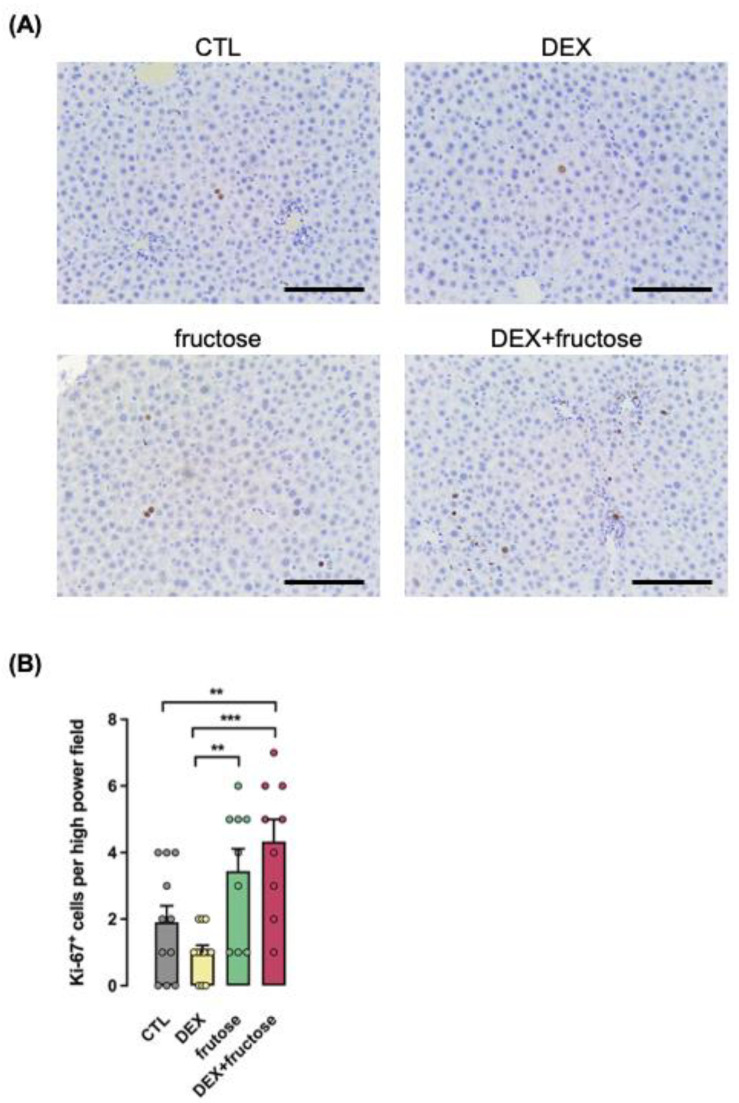
**The proliferation marker Ki-67 is increased in the liver of rats born to DEX-treated mothers and that chronically consumed fructose in adult life.** Representative images of liver sections used for immunohistochemical detection of Ki-67 (**A**). Average of Ki-67 positive cells (see Methods) are shown in (**B**). Data are presented as the mean ± SEM; ** *p* < 0.01 and *** *p* < 0.001 vs. the group indicated by the bracket. Horizontal bars = 10 μm. *n* = 9 to 11.

In addition to the markers mentioned above, the number of cells positive for Ki-67 were higher in the fructose–DEX but not in the fructose group when compared to CTL (126% higher; *p* < 0.01) (Figure 8).

## 4. Discussion

Epidemiological studies have already described that 11–13-year-old children born small for gestational age (SGA) display an increased prevalence of NAFLD [11,22]. Although the data correlating the prevalence of NASH with children born SGA are still contradictory [11,22], increased markers of liver damage and function were found to inversely correlate with birth weight in an older population of 60–79-year-old women [23]. Similarly, independent studies have clearly established a positive influence of fructose consumption and NAFLD in humans [24]. To the best of our knowledge, the present data reveal the unprecedented notion that low birth weight induced by in utero exposure to DEX interacts with fructose consumption during adult life to induce the expression/production of markers associated with steatohepatitis and HCC.

The results described herein are in accordance with our previous data showing that rats born to DEX-treated mothers exhibit exacerbated hepatic steatosis and glucose intolerance when treated with fructose during adulthood [15]. We therefore took advantage of this experimental model to explore whether this metabolic interaction was associated with reciprocal changes in molecular markers associated with steatohepatitis and risk for HCC.

Circulating AST levels are considered a nonspecific marker of NAFLD and NASH [25]. Previous data indicated that circulating AST levels do not correlate with birth weight in adult humans [23]. On the other hand, rats born with low birth weight due to maternal treatment with DEX were described to have increased circulating AST levels at the age of 12 weeks [26]. These data contrast the current findings showing that in utero exposure to DEX per se does not modulate AST levels in the offspring. Such a difference may owe to the distinct protocols of DEX treatment during pregnancy. While we used 0.1 mg/kg of DEX via drinking water, the protocol by Chen and colleagues consisted in subcutaneous injection of 0.2 mg/kg DEX [26].

Although neither exposure to DEX during neonatal life nor fructose treatment during adulthood interfered in AST levels, we found that rats exposed to both factors exhibited increased AST levels. Accordingly, we also found that increased hepatic MPO levels were an exclusive feature of rats exposed to both factors during their lives. It is relevant to point out that hepatic neutrophil infiltration is a crucial step that characterizes the transition from NAFLD to NASH, and increased MPO-positive cells comprise histological findings in NASH patients [27,28]. Furthermore, increased circulating MPO levels were reported in NASH but not in NAFLD patients [29]. Altogether, both increased hepatic MPO and AST levels indicate that fetal exposure to DEX and fructose consumption during adulthood lead to NASH in rats.

It is noteworthy that MPO levels also play a functional role in NASH. It was demonstrated that MPO-deficient mice are protected from NASH and fibrogenesis induced by a high-fat diet [29]. Growth arrest and DNA-damage-inducible 45a (Gadd45a) is also known as a crucial protein for the development of NASH, as evidenced by studies showing that Gadd45a-null mice display reduced steatohepatitis when subjected to a methionine- and choline-deficient diet [30]. Strikingly, Gadd45a also plays a key role by protecting HCC development in mice exposed to ultraviolet radiation and dimethylbenzanthracene [31]. One of the mechanisms by which Gadd45a controls cellular proliferation is by downregulating the PI3K/AKT pathway [32]. AKT signaling is classically recognized to stimulate cell proliferation, and its constitutive activation in HCC has been described by several studies [33].

Further evidence for the role of the AKT signaling pathway has emerged from studies focusing on TRIB3 (Tribbles Pseudokinase 3), a pseudokinase that interacts with AKT and inhibits its activity [34]. Inhibition of TRIB3 was described to enhance the tumorigenicity of several cancer cell lines by increasing AKT activity [35].

Remarkably, the above-mentioned pathways were modulated in the present study. While Gadd45a and Trib3 expression were reduced, AKT phosphorylation was increased in rats born to DEX-treated mothers and treated with fructose later in life. This set of data thus indicates that prenatal exposure to DEX and fructose consumption interact with each other to induce not only steatohepatitis, but also to stimulate molecular changes associated with HCC. In further support of this conclusion, we found increased Hspa4 and Hgf expression and reduced circulating IGF-1 exclusively in rats belonging to the DEX+fructose group. In this sense, it is important to highlight that Hspa4 and Hgf proteins are respectively increased in liver sections with HCC and in intrahepatic metastasis of HCC [36,37]. Circulating IGF-1, instead, is reduced in HCC patients [38].

Further relevance for the increased markers of HCC seen in the rats of the DEX+fructose group was, given by data on canonical proteins that mediate cell proliferation. Both Cyclin D1 and PCNA expression as well as the number of Ki-67 positive cells, were increased in the liver of rats exposed to DEX in utero and treated with fructose during adulthood. Of note, we previously demonstrated that the small intestine of DEX+fructose rats also shows a high proliferative epithelium, resembling the metabolic and morphological features of the neonatal jejunum phenotype [39].

It is important to emphasize that the present data are in line with previous investigations demonstrating that fructose *per se* does not induce HCC in wild-type strains of rodents. Fructose-induced HCC has only been detected in genetically modified prone strains or in mice exposed to nitrosamine injections [40]. In general, rodent studies have demonstrated that diet interventions lead to NASH-related alteration in most of the strains of mice, but consistent induction of HCC is only achieved if toxic compounds are combined (i.e., streptozotocin) [41].

Altogether, the present data indicate that rats subjected to a model of low birth weight and low relative liver weight develop molecular sings of non-alcoholic steatohepatitis and hepatocellular carcinoma when consuming excessive fructose during adulthood. Such findings are relevant to guide future epidemiological investigations aiming to determine whether this correlation is also true for the human species. If confirmed in a human context, the present data will serve as a basis for personalized early screening strategies or even preventive interventions in risk populations.

## Data Availability

The data presented in this study are available in article.
